# Supplementary material for: Immune-Related Endocrine Dysfunctions in Combined Modalities of Treatment: Real-World Data
Source: Cancers (Basel). 2021 Jul 28;13(15):3797. doi: 10.3390/cancers13153797 (PMC8345182; doi:10.3390/cancers13153797)
Supplement: Supplementary file 1 [file cancers-13-03797-s001.zip › cancers-1259884-supplementary.pdf]

cancers

MDPI

Supplementary

Immune-Related Endocrine Dysfunctions in Combined Modalities of Treatment: Real-World Data

Wing-Lok Chan et al.

Table S1. Targeted therapies used concurrently with immune-checkpoint inhibitors in this study.

Targeted therapy used	No. of patients
Total	54
Aflibercept	2
Axitinib	9
Avastin	7
Cabozatinib	1
Cetuximab	1
Dabrafenib, trametinib	1
Everolimus	1
Gefitinib	1
Lapatinib	1
Lenvatinib	19
Olaparib/ talazoparib	2
Ramucirumab	1
Regorafenib	1
Sorafenib	2
Temsirolimus	1
Trastuzumab, pertuzumab	4

Table S2. Number and percentage of Grade 3/4 toxicities.

G3/4 immune-related adverse effects	Total number of cases	ICI-alone *n* (%)	Dual-ICI *n* (%)	Chemo+ICI *n* (%)	Targeted+ICI *n* (%)
Acute renal impairment	3	2 (3.7%)	0 (0%)	0 (0%)	1 (14.3%)
Colitis/ Diarrhea	5	2 (3.7%)	1 (11.1%)	0 (0%)	2 (28.6%)
Deranged liver function	12	8 (14.8%)	2 (22.2%)	2 (18.2%)	0 (0%)
Infusional reaction	3	3 (5.6%)	0 (0%)	0 (0%)	0 (0%)
Immune thrombocytopenic purpura	3	3 (5.6%)	0 (0%)	0 (0%)	0 (0%)
Malaise	6	4 (7.4%)	0 (0%)	2 (18.2%)	0 (0%)
Neurological symptoms	1	0 (0%)	0 (0%)	1 (9.1%)	0 (0%)
Pancreatitis	1	0 (0%)	0 (0%)	1 (9.1%)	0 (0%)
Pneumonitis	11	7 (13%)	0 (0%)	4 (36.4%)	0 (0%)
Immune-related arthritis	7	6 (11.1%)	1 (11.1%)	0 (0%)	0 (0%)
Rashes	29	19 (35.2%)	5 (55.6%)	1 (9.1%)	4 (57.1%)
